# Effects of putrescine on the postharvest physiology characteristics in cowpea

**DOI:** 10.1002/fsn3.773

**Published:** 2019-01-28

**Authors:** Zhen Wang, Yunxiang Wang, Junyan Shi, Qiuli Zheng, Lipu Gao, Qing Wang, Jinhua Zuo

**Affiliations:** ^1^ Key laboratory of vegetable postharvest processing Ministry of Agriculture Beijing Key Laboratory of Fruits and Vegetable Storage and Processing Key Laboratory of Biology and Genetic Improvement of Horticultural Crops (North China) of Ministry of Agriculture Key Laboratory of Urban Agriculture (North) of Ministry of Agriculture Beijing Vegetable Research Center Beijing Academy of Agriculture and Forestry Sciences Beijing China; ^2^ College of Food Science and Engineering Ocean University of China Qingdao China; ^3^ Beijing Academy of Forestry and Pomology Sciences Beijing Academy of Agriculture and Forestry Sciences Beijing China; ^4^ Boyce Thompson Institute for Plant Research Ithaca NewYork

**Keywords:** cowpea, physiology characteristics, postharvest, putrescine, quality

## Abstract

The effects of putrescine (Put) treatment on postharvest physiology characteristics in cowpea during cold storage have been investigated. The results indicated that Put with 8 mmol/L treatment greatly delayed aging of the cowpea; the sensory quality of cowpea was well maintained; the increase in weight loss was also inhibited, and the decrease in the content of ascorbic acid, chlorophyll, and total phenol was reduced efficiently. Antioxidant enzyme activities containing POD, CAT, and APX were preserved at higher levels in treated groups than the control during cold storage. In addition, the activity of PPO was restrained with Put. Overall, the quality of cowpea was maintained by 8 mmol/L Put treatment during cold storage.

## 1 INTRODUCTION

Cowpea (*Vigna sinensis*) is widely cultivated in Asia, Africa, and Latin America in which they are planted more than 10 million ha of land (Nedumaran, Abinaya, Shraavya, Rao, & Bantilan, 2013). They are regarded as significant crops because of their high content of protein (20–35 g/100 g), high content of essential amino acid (e.g., lysine and threonine) which replenishes the lower lysine composition of grain (Cai, Hettiarachchy, & Jalaluddin, 2003; Phillips et al., 2003), and complex carbohydrates content, particularly starch and dietary fiber (Campos‐Vega, Loarca‐Piña, & Oomah, 2010). Cowpea is also rich in minerals such as Zn, Fe, Mg, and Ca (Sandberg, 2002). Besides, cowpea proteins are identified to be advantageous for curing diet‐induced hypercholesterolemia (Frota, Mendonca, Saldiva, Cruz, & Areas, 2008). Cowpea seeds could be eaten by cooking with other foods such as rice, steamed bread, pancake, and so on (Falade & Kolawole, 2013; Freire, 2012). However, the cowpea fruits are easy to decompose after harvest and cause worse quality (e.g., wilting in appearance) that makes marketing and circulation a tough issue. Thus, valid ways are essential to be taken to preserve the quality of cowpea in the process of transportation and storage.

Polyamines (PAs) are sort of Aliphatic amines with lower molecular weight, and the organic polycation metabolites would exist in nearly all living organisms (Valero, Martinez, & Serrano, 2002). Putrescine (Put), spermidine (Spd), and spermine (Spm) that are detected in plants are the main forms of PAs and have been reported to influence physiology of fruit after harvest (Perez et al., 2002). The usage of PAs could reduce rate of respiration, slow down ethylene production, inhibit senescence, retard color changes, induce mechanical resistance, improve fruit firmness, and reduce chilling injury of horticultural crops (Valero et al., 2002). Moreover, active outcome of PA on decreasing ethylene biosynthesis, which is a major predisposing factor in the maturation course, by restraining ACC synthase and transforming from ACC to ethylene has been vastly noted (Barman, Ram, & Pal, 2011; Koushesh, Arzani, & Barzegar, 2012). Moreover, prestorage Put treatment is widely mentioned to clearly inhibit ethylene causing and decelerate maturing in mango (Razzaq, Khan, Malik, Shahid, & Ullah, 2014); apricot (Koushesh et al., 2012); table grapes (Champa, Gill, Mahajan, & Arora, 2014); and cucumber (Qiao, Feng, Li, Zhang, & Liu, 2006). Hence, Put has a potential application on the part of postharvest treatments to keep good quality.

This study target was to evaluate the effects of 8 mmol/L putrescine solution treatment in the postharvest physiology characteristics of cowpea storage at 4 ± 1°C (day/night) to find out whether Put would keep the quality of cowpea effectively and whether these effects were related to the change of antioxidant enzyme system. The research might be useful for clarifying the physiological and biochemical effects in storage and preservation of cowpea. Meanwhile, it may offer a theoretical basis about exogenous Put treatment of cowpea.

## 2 MATERIALS AND METHODS

### 2.1 Cowpea material, treatments, and storage

Cowpeas (*V. sinensis,* “Qing‐jiang 18”) were harvested at commercial maturity from an organic orchard (110 m × 25 m) in Changping, Beijing. The pods were crisp, and grains were not exposed or slightly exposed with full green and no fibrosis. Then, all fruits were wrapped immediately in mesh bags which could prevent bruise and filled into foam boxes which were sealed to hold moisture. Whereafter, they were transferred into the laboratory quickly at ambient temperature within 2 hr. Fruits were chosen for the similar color, size, shape, firmness, and free from blemishes so as to ensure the uniformity of them. They were divided randomly into two groups with each group about 6.7 kg fruits. Respectively, immerse fruits for 10 min in (a) 8 mmol/L Put solution and (b) distilled water. After that, the fruits were dried for 1 hr at 25°C and 50% RH. Afterward, all fruits were sealed in polyethylene film bags of 0.03 mm in thickness with low moisture permeability, stored at 4 ± 1°C in dark, and kept 90%–95% relative humidity (RH). Each treatment group included three parallel controls, and a total of 14 kg fruits were applied at the first group of experiment. The shelf‐life was assessed daily with duplicate of 3 kg cowpea in each treatment.

The second group of tests, the properties of cowpea fruit after harvest treated by 8 mmol/L Put solution during storage at 4°C, was evaluated. Take the samples every 2 days and cut the fruit into small pieces. Soon after that, the fruit pieces were frozen in liquid nitrogen and stored at −80°C immediately. The frozen fruits samples were used to determine the content of ascorbic acid, chlorophyll, total phenolic, and the antioxidant activity of peroxidase (POD), catalase (CAT), ascrobate peroxidase (APX), and polyphenol oxidase (PPO). The first set of experiment was repeated three times from July to September in 2014. Besides, the second set of experiment was repeated three times from July to September in 2015.

Put was purchased from Kechuanghuida Biological Technology Co., Ltd. (Beijing, China). Prepare 1.5 L of 8 mmol/L Put solution and distilled water, respectively. The exogenous Put was dispersed into 1.5 L of distilled water, and another distilled water with same volume was used as control.

### 2.2 Sensory quality

Sensory quality in cowpea was measured using a little modification of the method of Gorny, Hess‐Pierce, and Kader (1999). All samples were evaluated visually by 10 judges at day 2, 4, 6, 8, and 10 of storage. Each treatment was coded and presented in random order and appraised based on general visual appearance and acceptability using this scale: 1 = poor (inedible); 3 = fair (limit of edible quality); 5 = good (limit of marketability); 7 = very good (freshness, hardness and juiciness); and 9 = excellent (fully marketability).

### 2.3 Weight loss

Weight loss (WL) was calculated based on the following formulas, using an electronic weighing scale with an accuracy of 0.5 g (UWA‐K‐015, Xiamen Andong Electronics Co., Ltd., Fujian, China) to record.

$$\text{WL}=\frac{\text{IW ‐ FW}}{\text{IW}}\times 100$$

With IW, initial fruit weight; and FW, final fruit weight after storage.

All measurements were taken in triplicate.

### 2.4 Ascorbic acid

The content of ascorbic acid was determined with the method of Jiang, Jahangir, Jiang, Lu, and Ying (2010). Fruit samples taken from fresh cowpea were passed through blender in the laboratory. Frozen tissue samples (2 g) from 30 fruits were blended with 80 ml of 5% meta‐phosphoric acid in a homogenizer and centrifuged. Then, 2 ml of the supernatant was poured in a 20‐ml test tube containing 0.1 ml of 0.2% 2,6‐DCIP sodium salt in water, 2 ml of 2% thiourea in 5% meta‐phosphoric acid, and 1 ml of 4% 2,4‐DNPH in 9 N sulfuric acid. After that, the mixtures were kept in a water bath at 37°C for 3 hr followed by an ice bath for 10 min. Following this, add five milliliters of 85% sulfuric acid and keep the mixtures at room temperature for 30 min before reading at 520 nm. The content of ascorbic acid was expressed as mg/g FW. Perform all of the measurements in triplicate.

### 2.5 Total chlorophyll

The content of total chlorophyll in cowpea was determined as detailed by Hasperué, Gómez‐Lobato, Chaves, Civello, and Martínez (2013). In each replicate, six fruits were mixed with 25 ml 80% (v/v) acetone and centrifuged at 10,000 g for 10 min at 4°C. The chlorophyll content in the supernatant was determined by a Hitachi U‐1800 spectrophotometer (Hitachi High‐Technologies Corp., Tokyo, Japan). The content of chlorophyll was expressed as mg/g FW. Perform all of the measurements in triplicate.

### 2.6 Total phenolic

The content of total phenolic was determined by the description of Ainsworth and Gillespie (2007). Frozen tissue and flesh tissue were ground in a mill and approximately 2 g from three different fruits were added to 6 ml ethanol, respectively. Swirl the suspension liquid up to 12,000 *g* centrifugation for 20 min at 4°C by frozen centrifuge. Save the supernatant and add water up to 100 ml. Two hundred microliters of 1:1 Folin–Ciocalteu reagent was added into test tubes containing 400 μl of ethanolic extracts and 800 μl of water. Samples were vortexed, at 20°C after 3 min, and 400 μl of 20% (w/v) Na_2_CO_3_ in 0.1 mol/L NaOH was added. After that, fill each test tube with 3 ml water, vortexing and incubated at 20°C for 1 hr. Measure the absorbance at 760 nm and calculate the content of total phenolic compounds using phenol as a standard. Repeat the experiment three times, and each treatment with three replicates.

### 2.7 The activity of PPO, POD, CAT, and APX

Take 2 g of frozen tissue samples from each fruit and homogenized them at 4°C in 10 ml of the appropriate ice‐cold extraction buffers which contains 0.2 g of polyvinylpolypyrrolidone (PVPP). The extraction buffer used for the assay of PPO and POD was 100 mmol/L sodium phosphate (pH 6.4). In addition, for the assay of CAT, the extraction buffer was 100 mmol/L sodium phosphate (pH 7.8). Centrifuge the homogenate under 12,000 g for 20 min in 4°C, and the supernatant was prepared for the enzyme assay.

PPO and POD activities were analyzed as explained by Liu, Tian, Meng, and Xu (2007). Measure the activity of PPO by adding 50 μl enzyme preparation to 3.0 ml of catechol substrate (500 mmol/L) and test the absorbance at 410 nm immediately over 1 min. The variation in the absorbance of the reaction at 410 nm was fixed every 5 s. One unit of activity was determined as the amount of enzyme required to cause an increase of one absorbance unit at 410 nm in 1 min. Using guaiacol as substrate, POD activity was determined. The reaction mixture consisting of 1 ml of crude extract, 2 ml of guaiacol (8 mmol/L), was incubated for 30 min at 30°C. After 1 ml of H_2_O_2_ (24 mmol/L) was added, the increase in absorbance at 470 nm over 1 min was determined. The change in the absorbance of the reaction solution at 470 nm was determined every 5 s. One activity unit was ensured as the amount of enzyme required as an increase of one absorbance unit at 470 nm within 1 min. PPO and POD activities were defined as a change in OD_470_ per gram FW per minute. Perform all of the measurements in triplicate.

The activity of CAT was measured with detailed by Ali, Hahn, and Peak (2005). Take 3 ml reaction solution of CAT which contained 1.9 ml of 100 mmol/L sodium phosphate buffer with the pH 7.8, 1 ml of 0.3% H_2_O_2_, and 0.1 ml enzyme extract. By adding the enzyme extract, the reaction started. The tendency in the absorbance of the reaction solution at 240 nm was measured every 5 s. Define each unit of activity the amount of enzyme required to render a decrease of 0.01 absorbance unit at 240 nm/min. Each treatment did three replicates.

The activity of APX was analyzed by calculating the decrease in absorbance of ascorbic acid (AsA) at 290 nm (Zhang, Hu, & Yao, 2010). The reaction mixture contained 2.6 ml of PBS (pH 7.5, containing 0.1 mmol/L EDTA and 0.5 mmol/L AsA), 0.1 ml of sample, and 0.3 ml of 2 mmol/L H_2_O_2_.

### 2.8 Statistical analysis

Each measurement was performed by the SPSS version 13.0 statistical software (SPSS Inc., Chicago, IL, USA). The data were subjected to one‐way ANOVA. Duncan's multiple range tests were also used to determine the difference of means from the ANOVA. It was considered to be significant variation when differences were at *p* < 0.05. There were no significant interactions between treatments and experiments, and the data provided in this study are a collection of three independent repetitive experiments.

## 3 RESULTS

### 3.1 Sensory quality evaluation in cowpea with Put treatment

**Figure 1:**
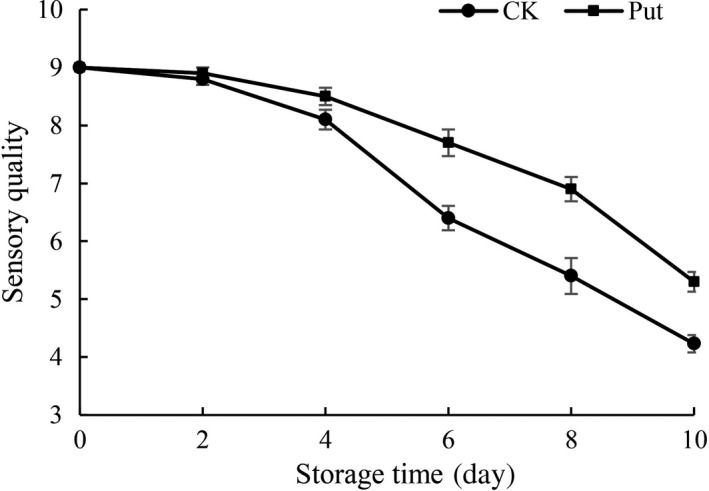
Effect of Put treatment on sensory quality of cowpea stored at 4°C. Each value is presented as the mean ± *SE* (*n* = 10). The differences among the treatments indicated with the same letter vertically were not significant according to Duncan's multiple range test at *p* < 0.05

The sensory quality of cowpea fruits which were stored at 4°C for 10 days and 80 ± 5% RH is presented in Figure 1. The control group demonstrated clear dehydration phenomenon and an evident decrease in the sensory quality point‐scoring system during the 10‐day storage and observation. The scores changed from 9 to 4.23; while Put decelerates the decrease and demonstrated 5.17 scores when treated with 8 Mm Put on fruits until the 10‐day storage. Moreover, it is shown that the control group reached a score of 5.13 in the sensory quality on the 8th day. In general, the sensory quality of cowpea fruits which were treated in 8 mmol/L Put could become over the period of storage at 4°C, relative to untreated fruits.

### 3.2 Effect of Put treatment on weight loss of cowpea

**Figure 2:**
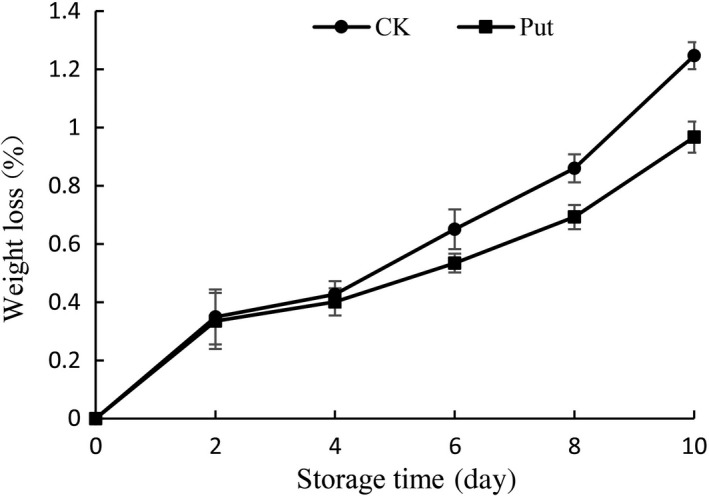
Effect of Put treatment on weight loss of cowpea stored at 4°C. Each value is presented as the mean ± *SE* (*n* = 3). The differences among the treatments indicated with the same letter vertically were not significant according to Duncan's multiple range test at *p* < 0.05

As shown in Figure 2, Put‐treated fruits showed a slower weight loss than that of untreated fruits although there were growing trends in all test groups during the experiment process. The loss of weight on the 10th day of control group was 1.31 times higher than in 8 mmol/L Put‐treated fruits, and significant variations were shown in all treated tests (*p *< 0.05).

### 3.3 Ascorbic acid determination in cowpea with Put treatment

**Figure 3:**
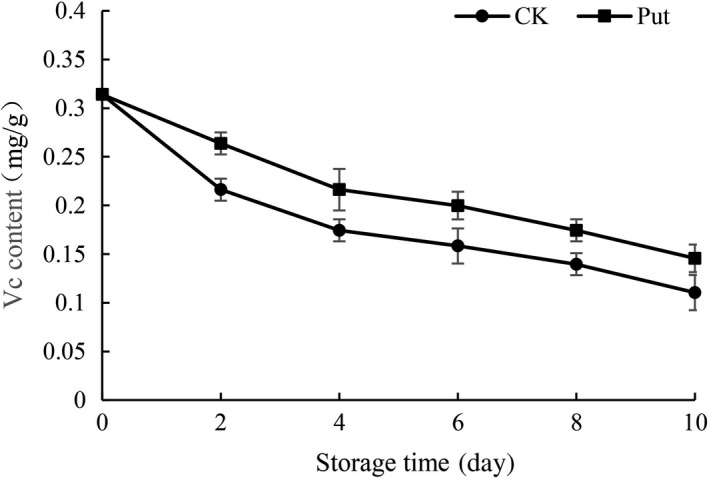
Effect of Put treatment on Vc content of cowpea stored at 4°C. Each value is presented as the mean ± *SE* (*n* = 3). The differences among the treatments indicated with the same letter vertically were not significant according to Duncan's multiple range test at *p* < 0.05

The variation on ascorbic acid content is indicated in Figure 3. The content of ascorbic acid dropped notably during the storage of 10 days in all the samples. However, the application of Put significantly slowed the falling tendency, and notable variation was founded in the treated groups with 8 mmol/L Put and the control groups (*p* < 0.05).

### 3.4 Chlorophyll content analysis in cowpea with Put treatment

**Figure 4:**
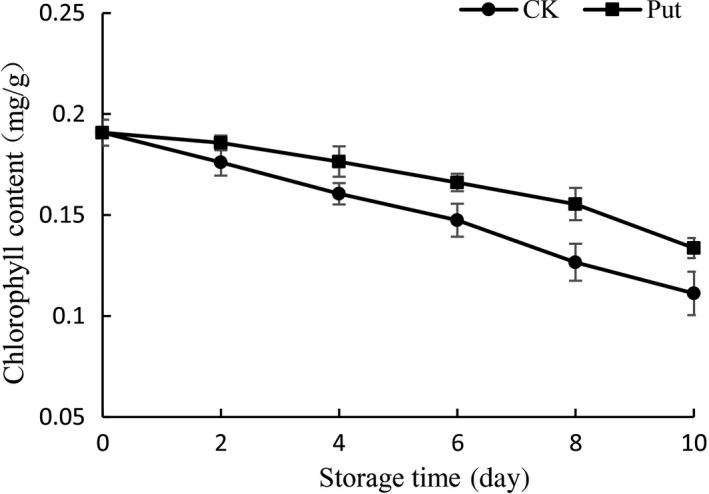
Effect of Put treatment on chlorophyll content of cowpea stored at 4°C. Each value is presented as the mean ± *SE* (*n* = 3). The differences among the treatments indicated with the same letter vertically were not significant according to Duncan's multiple range test at *p* < 0.05

The effect of Put treatment in cowpea total chlorophyll content was examined in the range of 0–10 days (Figure 4). The degree of reduction of chlorophyll content, as shown in Figure 4, was lower in Put‐treated groups than in the control group. Up to the 10th day, there was significant variation between Put treatment samples and the control group when the total chlorophyll content in Put treatment samples had been reduced by 27.78% relative to the initial content of chlorophyll; however, the control group demonstrated it had decreased by 36.11% in contrast with the initial data of chlorophyll content.

### 3.5 Total phenolic content analysis in cowpea with Put

**Figure 5:**
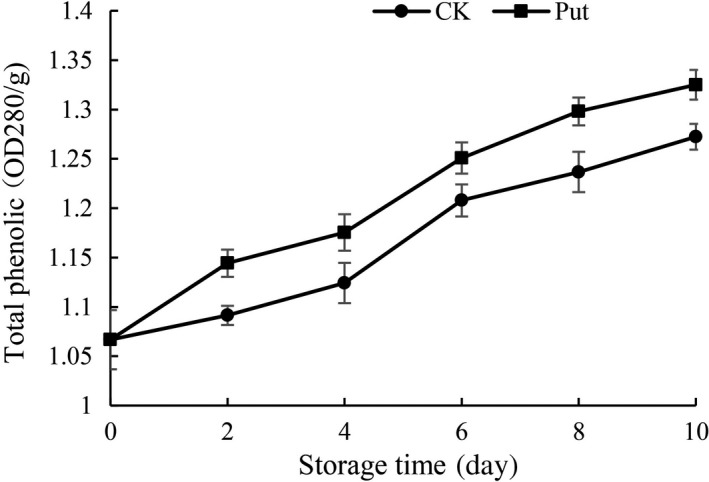
Effect of Put treatment on total phenolic content of cowpea stored at 4°C. Each value is presented as the mean ± *SE* (*n* = 3). The differences among the treatments indicated with the same letter vertically were not significant according to Duncan's multiple range test at *p* < 0.05

In all fruits, the level of total phenolic content increased during storage regardless of treatment (Figure 5). There are significant differences between the Put‐treated fruit and the control group. Put‐treated samples displayed higher data of total phenolic content compared to the control group among all data. On the 10th day of the storage, the total phenolic content with 8 mmol/L Put‐treated fruit was higher than the control group.

### 3.6 PPO activity determination in cowpea with Put treatment

**Figure 6:**
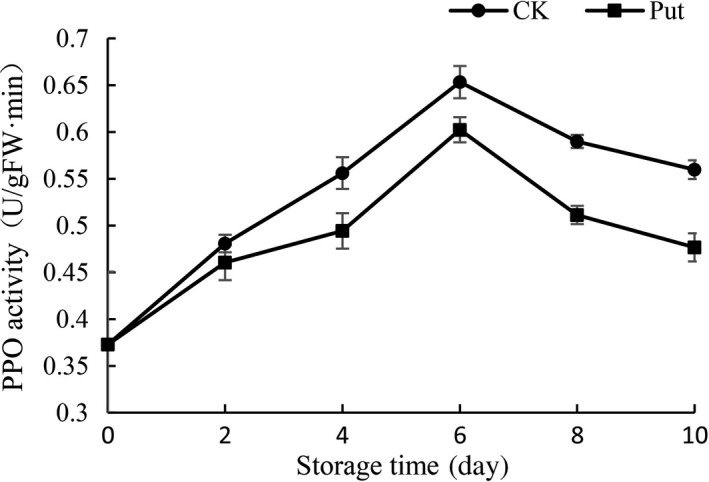
Effect of Put treatment on PPO activity of cowpea stored at 4°C. Each value is presented as the mean ± *SE* (*n* = 3). The differences among the treatments indicated with the same letter vertically were not significant according to Duncan's multiple range test at *p* < 0.05

The data of cowpea with Put treatment showed that the PPO activity had a notable growth at the 2‐day storage, and it came to the maximum at the 6th day (Figure 6). The 8 mmol/L Put treatment significantly inhibited PPO activity after fruits were harvested, relative to the control group (*p* < 0.05).

### 3.7 POD activity determination in cowpea with Put treatment

**Figure 7:**
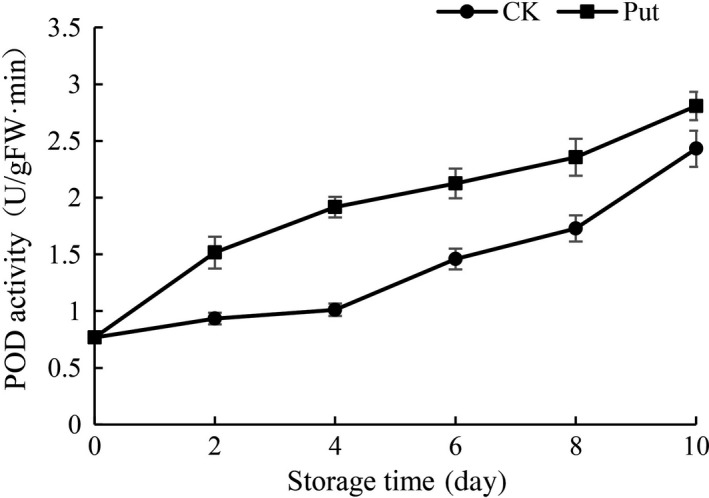
Effect of Put treatment on POD activity of cowpea stored at 4°C. Each value is presented as the mean ± *SE* (*n* = 3). The differences among the treatments indicated with the same letter vertically were not significant according to Duncan's multiple range test at *p* < 0.05

The activity of POD steadily increased in the Put‐treated samples and control during 10‐day period of storage at 4°C (Figure 7). In Put‐treated samples, the activity of POD was much higher than that in the control group, peculiarly on the 8th and 10th days. It was obvious that the degree of activity of POD was greatly higher at the 8th day and the 10th day compared to Put‐treated groups with the control group.

### 3.8 CAT activity determination in cowpea with Put treatment

**Figure 8:**
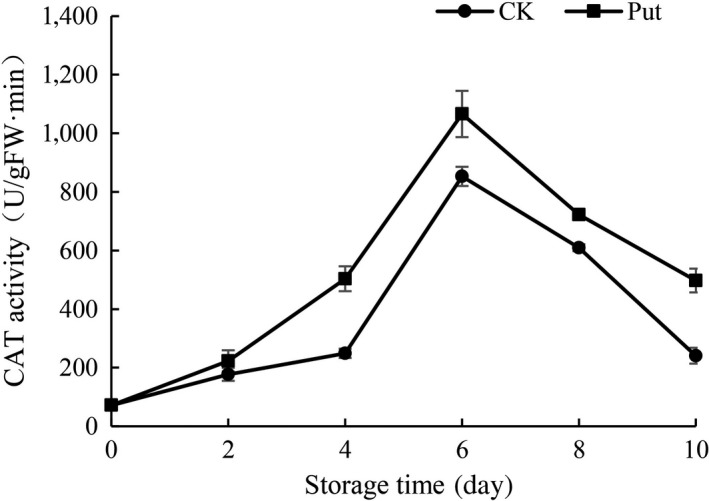
Effect of Put treatment on CAT activity of cowpea stored at 4°C. Each value is presented as the mean ± *SE* (*n* = 3). The differences among the treatments indicated with the same letter vertically were not significant according to Duncan's multiple range test at *p* < 0.05

An increase in CAT activity of cowpea was characterized within the earlier 6 days in the storage of 4°C in both Put‐treated groups and untreated group and arrived at the supreme at the 6th day in all groups. Subsequently, the trend began to decrease after the 6th day in each group (Figure 8). However, the top level of CAT activity was usually appeared in 8 mmol/L Put‐treated groups, relative to the untreated group during the storage.

### 3.9 APX activity determination in cowpea with Put treatment

**Figure 9:**
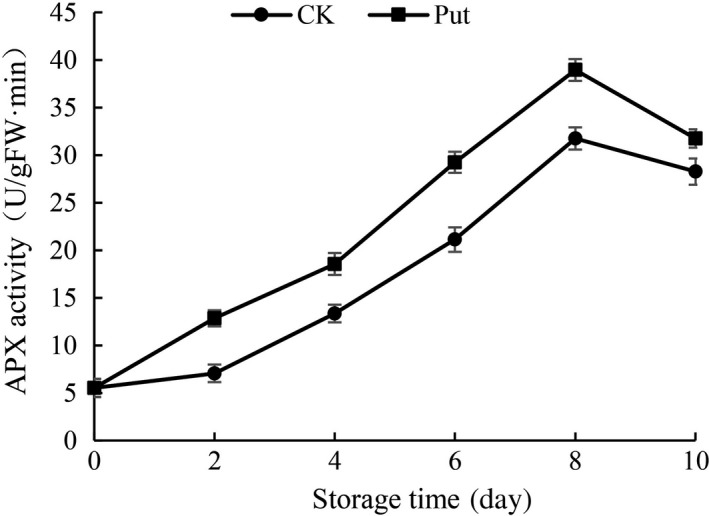
Effect of Put treatment on APX activity of cowpea stored at 4°C. Each value is presented as the mean ± *SE* (*n* = 3). The differences among the treatments indicated with the same letter vertically were not significant according to Duncan's multiple range test at *p* < 0.05

The influence of Put‐induced change in the activity of APX is shown in Figure 9. The activity of APX in cowpea decreased after 8‐day storage in both Put‐treated and untreated groups over the storage period, while the Put treatment with 8 mmol/L clearly induced APX activity, relative to the control group (*p* < 0.05).

## 4 DISCUSSION

Maintaining higher organoleptic qualities by putrescine has been observed in grapes (Shiri, Ghasemnezhad, Bakhshi, & Sarikhani, 2013), mango (Jawandha, Gill, Singh, Gill, & Singh, 2012), and strawberry (Khosroshahi, Esna‐ashari, & Ershadi, 2007). In this study, postharvest soaking treatment of putrescine resulted in better organoleptic quality (Figure 1). Moreover, application of Put reduced fruit weight loss during ripening as well as cold storage, which may be ascribed to consolidation or stabilization of both cell integrity and tissue permeability (Mirdehghan et al., 2007). During storage, higher rates of weight loss were observed in control fruit, while in Put‐treated fruits, the weight loss was significantly suppressed by exogenous treatment of putrescine (Figure 2). Similarly, prestorage dips of Put also reduced weight loss in mango (Malik & Singh, 2005). In our study, Put treatment with 8 mmol/L has an effective role in delaying weight loss and maintaining sensory quality of cowpea in contrast with the control group (Figures 1 and 2). This result may also attribute to the low levels of ethylene production after treatment with putrescine (Serrano, Martinez‐Romero, Guillen, & Valero, 2003).

Nutritional components in cowpea fruits are the most important indexes to access the quality of cowpea. Plant senescence is always accompanied with the change of color, which is related to the degradation of chlorophyll (Aiamlaor, Nakajima, Shigyo, & Yamauchi, 2012). The chlorophyll contents are implied by the apparent color of cowpea. Moreover, people often decide whether to buy or not based on color of fruit. During this study, with the advancement of storage in cowpea fruit, a decreasing trend on aspects of chlorophyll content and Vc took place in each group similarly (Figures 3 and 4). Nevertheless, the downtrend with Put treatment was significantly delayed for the exogenous application of Put, which was in accordance with the study on mango (Razzaq et al., 2014). Additionally, Ali, Muhammad, Sijam, and Siddiqui (2011) revealed that the delay in color transition may be due to the low speed of respiration as well as diminished ethylene production so that the internal atmosphere of the fruit could be modified. The cowpea fruit treated with 8 mmol/L Put displayed relatively less attenuation. These results may be due to the Put treatment retarding the senescence procedure. In addition, Noctor and Foyer (1998) reported that Vc was a crucial antioxidant among plants which could be used to scavenge reactive oxygen species (ROS) in direct ways. Related enzymes could combine together to regulate the degree of reaction. The higher content of Vc could reveal the finer oxidation resistance in Put treatment groups (Han et al., 2014).

The previous literature reported that the susceptibility to senescence in the internal structure may be a correlative factor which would influence the total phenolic content (Massolo, Concellón, & Chaves, 2011). The decrease in total phenolic content could be attributed to the senescence caused by the disintegration of cell construction (Razzaq et al., 2014). In this study, irrespective of treatments, the content of total phenolic demonstrated an upward trend with increase in the storage period, while this increase was significantly higher in Put‐treated fruits (Figure 5). During storage, the increase in the level of total phenolic contents may be due to senescence through breakdown of cell structure (Ghasemnezhad, Shiri, & Sanavi, 2010). The effect of Put treatment to maintain total phenolic contents could be ascribed to the ability to delay the senescence process in treated fruit. The results also indicated that the antioxidants may also increase along with an increase of total phenolic contents. Thus, it might be concluded that total phenolic contents are directly linked and correlated with the antioxidant activity. Previously, a positive correlation among total phenolic and antioxidant activity has also been reported in apricot (Ghasemnezhad et al., 2010) and mango (Palafox‐Carlos et al., 2012). In another aspect, PPOs accelerate the reaction of the oxidation of phenolic to quinones taking advantage of oxygen as the ultimate electron acceptor; however, hydrogen peroxide is the terminal electron acceptor for peroxidases. When quinone was produced, the accumulation of melanin‐like pigments is caused by secondary nonenzymatic reactions, bringing a brown bad appearance to the organization (Massolo et al., 2011). Our results showed that the changing tendency of the PPO activity was in close agreement with the previous research reported by Concellón and Añón (2004). Anyway, Put‐treated fruit could control the rising trend of PPO activity in the period of 10‐day storage (Figure 6). The activity of PPO was usually evidently lower in Put treatment group compared with untreated group in this research.

Oxidative stress could be inhibited by inner antioxidant enzyme system which are crucial compositions in cellular protection system and make effects on preventing plants from hurt caused by the accumulation of ROS (Duan, Li, Guo, & Kang, 2008; Groppa, 2008; Rider et al., 2007; Zhou, Yu, Mao, Huang, & Song, 2006). In this research, the activity of CAT was remarkably higher in 8 mmol/L Put treatment groups as compared to controls during the period of cold storage (Figure 8). CAT being the most important antioxidative enzyme plays a key role in ROS control, specifically H_2_O_2_. Increased activity of CAT is a crucial to defensive mechanisms against ROS injuries, whereas lower CAT activity suggests a weakened capability of cells to scavenge H_2_O_2_ (Ng et al., 2005). Similarly, Put applications significantly increased the activity of CAT in “Asgarabadi” and “Bagheri” apricots (Saba, Arzani, & Barzegar, 2012). Our results also showed a higher activity of APX in the treated fruit during 10 days in contrast with control fruit, as well as much higher activity of CAT and POD in Put‐treated groups (Figures 7, 8 and 9). The increase in antioxidant enzymes may be due to Put binding to antioxidative enzymes, or conjugated to small antioxidant molecules and their permeation to the oxidative stress sites in the cells. Increase in antioxidant enzyme activity in Put‐treated fruits may provide protection for biomolecules against oxidative damage caused by ROS, especially the O^2−^ radical (Verma & Mishra, 2005). Likewise, the current research also suggested that Put‐treated fruits markedly promoted activities of POD and CAT in mango fruit (Razzaq et al., 2014). Thus, the treatment groups with Put solution would give full play in removal ability of H_2_O_2._

Overall, the results revealed that postharvest application of 8 mmol/L Put treatment maintained better quality of cowpea fruits during the period of storage. The data acquired demonstrated that the use of Put treatment with 8Mm displayed a good score of sensory quality, significantly inhibited the downtrend of weight loss in fruits, as well as reducing the downward trend in aspects of ascorbic acid, chlorophyll, and total phenol. PPO activity was inhibited by Put treatment. Besides, the activities of POD, CAT, and APX of cowpea were induced greatly during the cold storage of 10 days, relative to controls. Consequently, the application with Put may be one effective way to keep postharvest quality of cowpea. Furthermore, molecular mechanism is needed for later studies. Alternatively, Put combined with other treatments may be used as a potential agent in the probable functional mechanism so as to improve shelf‐life after harvesting.

## CONFLICT OF INTERESTS

The authors declare no conflict of interest.
